# Prospective evaluation of contrast-enhanced ultrasound of breast BI-RADS 3–5 lesions

**DOI:** 10.1186/s12880-020-00467-2

**Published:** 2020-06-17

**Authors:** Eva Janu, Lucie Krikavova, Jirina Little, Karel Dvorak, Dagmar Brancikova, Eva Jandakova, Tomas Pavlik, Petra Kovalcikova, Tomas Kazda, Vlastimil Valek

**Affiliations:** 1 The Clinic of Radiology and Nuclear Medicine, The University Hospital Brno, Brno, Czech Republic; 2 The Clinic of Radiology and Nuclear Medicine, Faculty of Medicine, Masaryk University, Brno, Czech Republic; 3 The Clinic of Internal Medicine – Haematology and Oncology, The University Hospital Brno, Brno, Czech Republic; 4 The Institute of Pathology, The University Hospital Brno, Brno, Czech Republic; 5 The Institute of Biostatistics and Analyses, Faculty of Medicine, Masaryk University Brno, Brno, Czech Republic; 6 Department of Radiation Oncology, Masaryk Memorial Cancer Institute, Zluty kopec 7, 65653 Brno, Czech Republic; 7 Department of Radiation Oncology, Faculty of Medicine, Masaryk University, Brno, Czech Republic

**Keywords:** Contrast ultrasonography, CEUS, Breast cancer, TI curve, Ultrasound

## Abstract

**Background:**

To determine the benefit of contrast-enhanced ultrasound (CEUS) in the assessment of breast lesions.

**Methods:**

A standardized contrast-enhanced ultrasound was performed in 230 breast lesions classified as BI-RADS category 3 to 5. All lesions were subjected to qualitative and quantitative analysis. MVI (MicroVascular Imaging) technique was used to derive qualitative analysis parameters; blood perfusion of the lesions was assessed (perfusion homogeneity, type of vascularization, enhancement degree). Quantitative analysis was conducted to estimate perfusion changes in the lesions within drawn regions of interest (ROI); parameters TTP (time to peak), PI (peak intensity), WIS (wash in slope), AUC (area under curve) were obtained from time intensity (TI) curves. Acquired data were statistically analyzed to assess the ability of each parameter to differentiate between malignant and benign lesions. The combination of parameters was also evaluated for the possibility of increasing the overall diagnostic accuracy. Biological nature of the lesions was verified by a pathologist. Benign lesions without histopathological verification (BI-RADS 3) were followed up for at least 24 months.

**Results:**

Out of 230 lesions, 146 (64%) were benign, 67 (29%) were malignant, 17 (7%) lesions were eliminated. Malignant tumors showed statistically significantly lower TTP parameters (sensitivity 77.6%, specificity 52.7%) and higher WIS values (sensitivity 74.6%, specificity 66.4%) than benign tumors. Enhancement degree also proved to be statistically well discriminating as 55.2% of malignant lesions had a rich vascularity (sensitivity 89.6% and specificity 48.6%). The combination of quantitative analysis parameters (TTP, WIS) with enhancement degree did not result in higher accuracy in distinguishing between malignant and benign breast lesions.

**Conclusions:**

We have demonstrated that contrast-enhanced breast ultrasound has the potential to distinguish between malignant and benign lesions. In particular, this method could help to differentiate lesions BI-RADS category 3 and 4 and thus reduce the number of core-cut biopsies performed in benign lesions. Qualitative analysis, despite its subjective element, appeared to be more beneficial. A combination of quantitative and qualitative analysis did not increase the predictive capability of CEUS.

## Background

Breast cancer is statistically the most common malignant disease in females. In 2016 the incidence was 77.49 and mortality 14.09 per 100,000 women according to the Czech National Cancer Registry [1]. Despite increasing incidence rates, mortality rates have been stable or slightly decreasing since the mid-1990s [1]. This favorable trend can be attributed to early diagnosis of breast cancer due to the screening program and to more effective therapy [2]. Ultrasound and mammography are the standard imaging techniques for detection and classification of breast lesions. However, conventional ultrasound is not sufficient to determine the biological type of a lesion in many cases [3]. Contrast-enhanced ultrasound (CEUS) is a modern method which allows real-time evaluation of perfusion changes and the micro-vascular architecture of a lesion with greater accuracy than conventional Doppler mapping [4]. CEUS provides extra information about a lesion in addition to the B-mode characteristics (position, size, echogenicity and boundary) and Doppler imaging of the supplying vessels. This examination can only be performed with an ultrasound machine which allows detection of the contrast agent with sufficient sensitivity.

Increasing prevalence of breast cancer patients is currently causing an increasing workload of screening centers. As a consequence, availability of experienced radiologists may be the main cause of insufficient screening services for our patients in the near future. Modern artificial intelligence algorithms capable to facilitate objective image analysis will facilitate, above all, the evaluation of mammographic images, however, the subjective real-time evaluation of ultrasound examination will remain in the hands of the physician. One may assume, that with the mentioned radiologist shortage, it will be necessary to facilitate their expertise especially in this area. In addition to cutting-edge research of novel imaging approaches, investigation focused on to more available methods has the potential to contribute to more valid evaluation of lesions in large numbers of patients who would not otherwise access to modern methods such as MR. Facing more and more limited capacity of our breast screening center owing to increasing prevalence, we decided to reevaluate consecutive patients CEUS examination performed in prospective fashion couple of years ago by single provider and, thus, provide another comparative set for other physicians within their learning curve of this subjective method. The aim of this prospective study is to evaluate the value of contrast-enhanced ultrasound and its implementation into the diagnostic algorithm of breast cancer.

## Methods

### Patients selection

Consecutive patients referred for breast ultrasound examination of their solid breast lesions between September 2012 and December 2014 were assessed for eligibility in this prospective study. All patients were examined and diagnosed at our specialized ultrasound outpatient department at The University Hospital Brno, Czech Republic which focuses on breast imaging. A standardized BI-RADS™ (Breast Imaging Reporting and Data System) Classification System [5] was used to evaluate the findings on the conventional ultrasound examination. Lesions of BI-RADS category 3 to 5 (category 3: probably benign, category 4: suspicious abnormality and biopsy should be considered, category 5: highly suggestive of malignancy) were eligible for this study.

### CEUS examination

All examinations were performed by one physician according to a standardized protocol approved by the Ethics Committee of the University Hospital Brno. An informed consent form was signed by each patient before the examination. Ultrasound examinations were performed on the Philips iU22 ultrasound machine using the L12–5 high-frequency line transducer. All lesions were examined after the administration of a contrast agent. In order to keep a constant position of the probe during the examination, a dual display of grayscale and contrast enhanced image was used to allow simultaneous visualization. The plane with the longest lesion diameter was selected as a reference scan. In addition to the stable position of the transducer during the examination, the minimum compression of the target area was retained. Each time, a bolus injection of 2.5 ml of the contrast agent SonoVue® (Braco) was administered into a vein followed by 10 ml saline flush. The acquired videos, at least 60 s “examination loops”, displaying perfusion changes in the region of interest (ROI), were further processed with Qlab® Quantification software version 8.0 (Philips). Both qualitative and quantitative analysis were employed for evaluation of acquired data.

Gray scale and contrast enhanced ultrasound were followed by core-needle biopsy of lesions BIRADS 4 and 5. Histopathological assessment was conducted by a pathologist specialized in breast cancer. Benign lesions without histopathological verification (BI-RADS 3) were followed up for at least 24 months in order to confirm their benign status.

### CEUS analysis

**Fig. 1:**
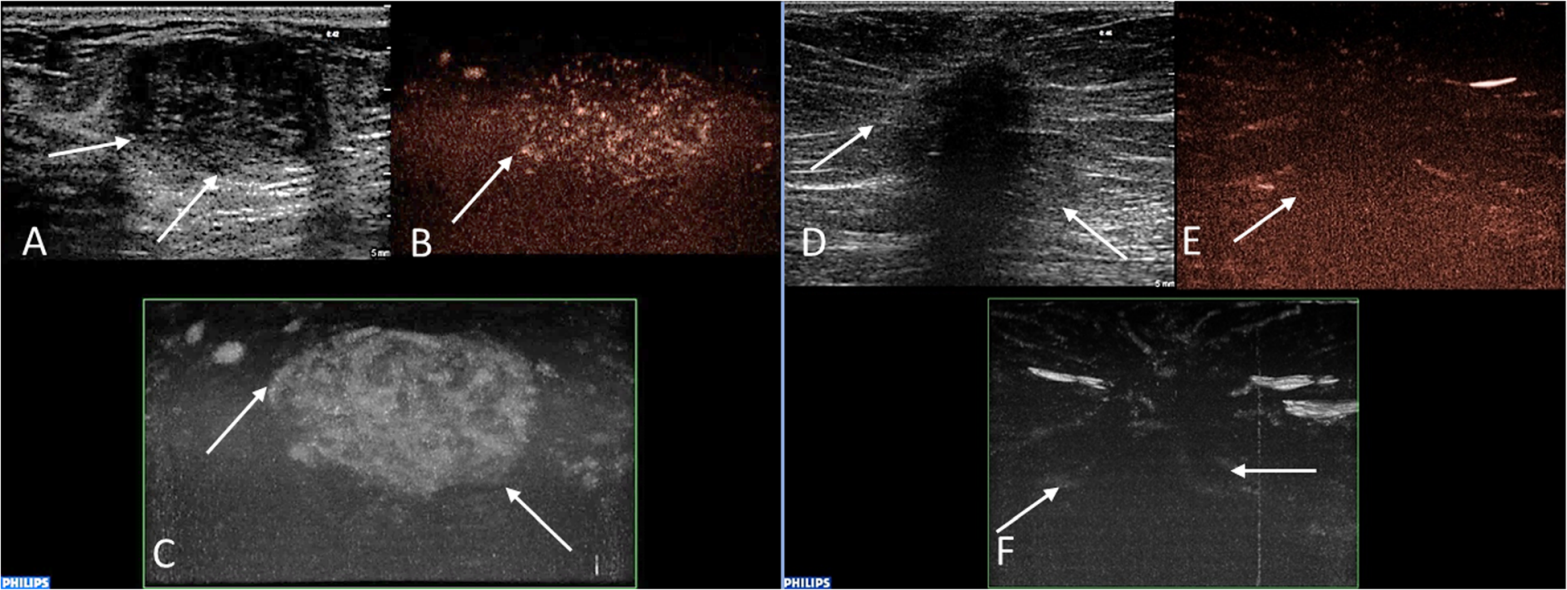
Examples of the evaluation of lesions´ vascularity using the MVI technique. Histopathologically verified fibroadenoma in a 37-year-old female patient (**a**, **b**, **c**). There is a well-circumscribed hypoechoic mass (**a**) with heterogenous internal enhancement (**b**) and with rich vascularization compared to the surrounding tissue (**c**). Verified invasive carcinoma of no special type (NST) in a 79-year-old female patient (**d**, **e**, **f**). Greyscale ultrasound shows poorly defined hypoechoic mass (**d**). A post-processed CEUS image using MVI application displays peripheral penetrating vessels (**e**) and heterogeneous internal perfusion of the malignant tumor (**f**). Abbreviations: MVI, MicroVascular Imaging; NST, invasive carcinoma of no special type; CEUS, contrast-enhanced ultrasound

**Fig. 2:**
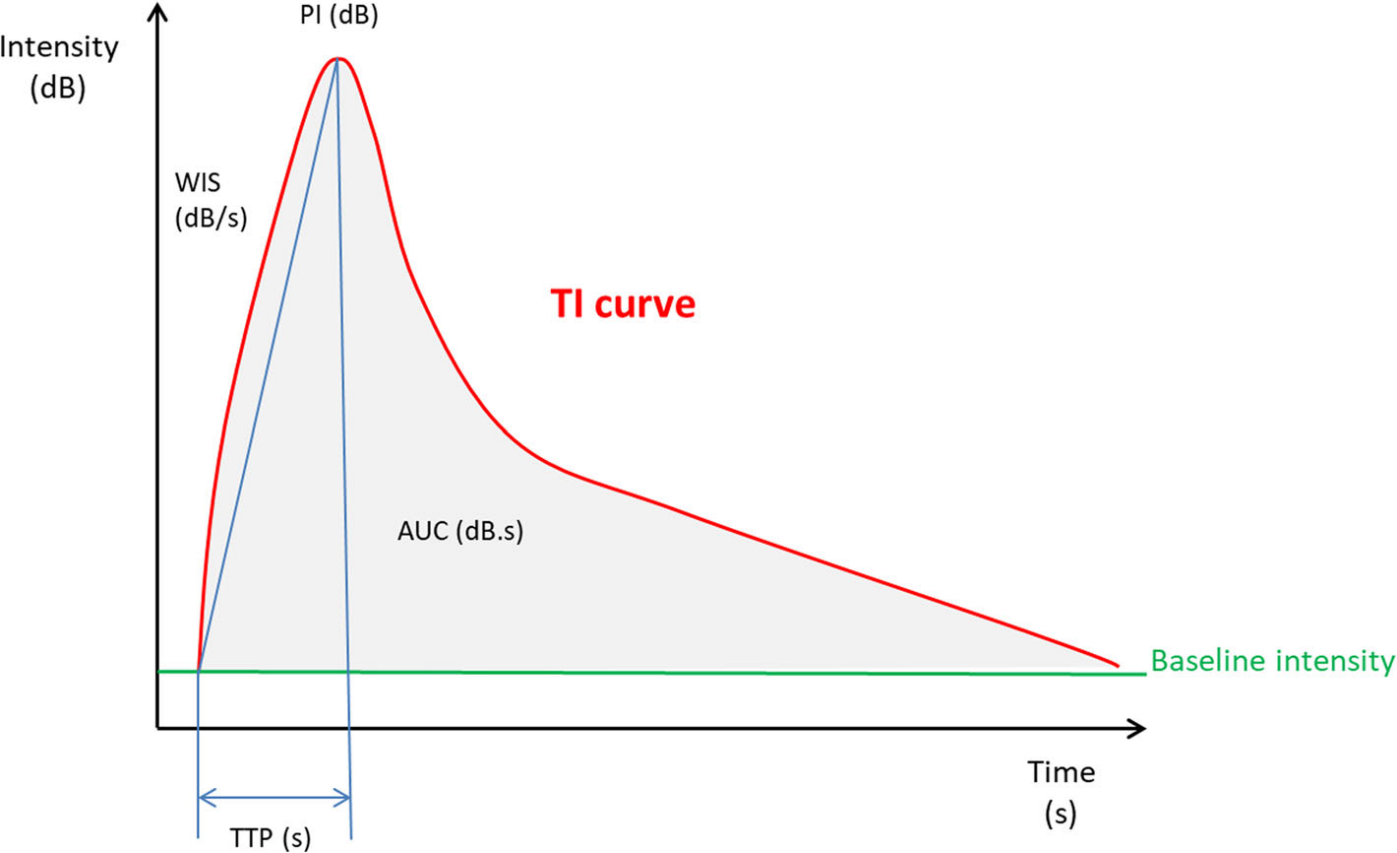
A model time-intensity curve with marked quantitative parameters and their values. Abbreviations: TI, time intensity curves; PI, peak intensity; WIS, wash in slope; AUC, area under curve; TTP, time to peak

**Fig. 3:**
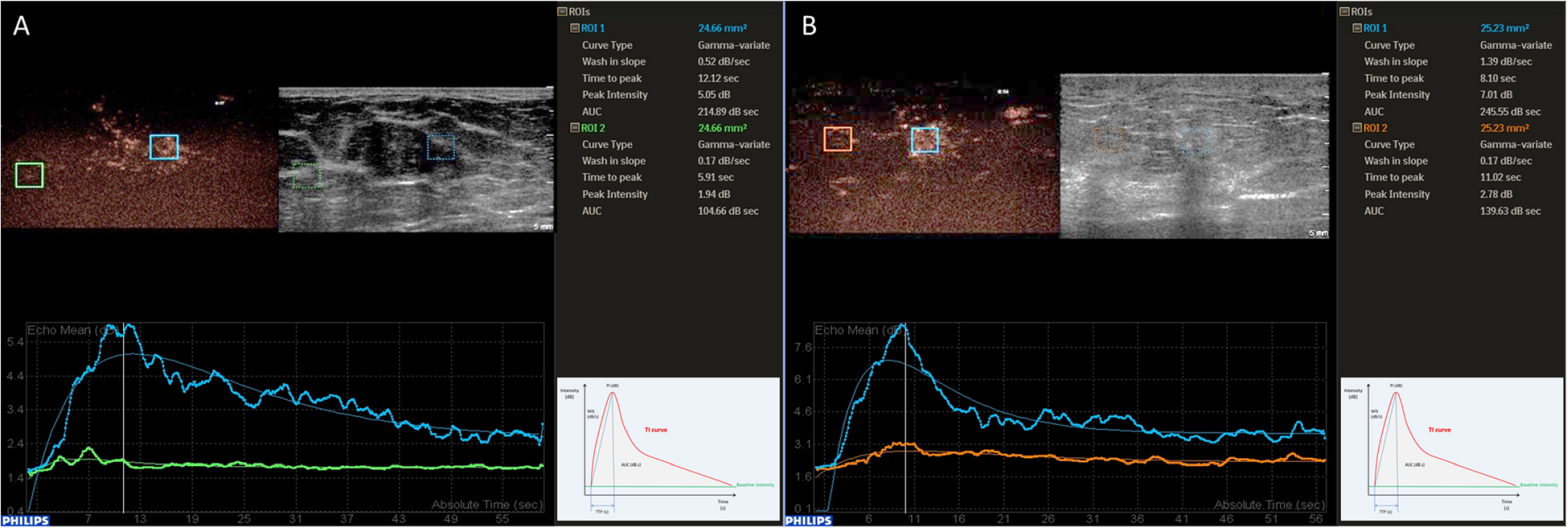
Examples of quantitative data acquisition using ROI. Fibroadenoma in a 39-year-old female (**a**). Strikingly rich vascularization of the lesion (blue ROI) compared to minimal blood perfusion in surrounding breast tissue (green ROI). Gradual enhancement and a gradual wash out of contrast agent (corresponding TI curves below). Invasive carcinoma of no special type (NST) in a 59-year-old female (**b**) with Pronounced enhancement of the lesion and adjacent tissue (blue ROI), the lesion is ill-defined. After fast enhancement of the tumor, early wash-out can be observed (corresponding TI curve below). The enhancement of surrounding breast tissue is (orange ROI, corresponding TI curve below). Abbreviations: ROI, region of interest; TI, time intensity curves; NST, invasive carcinoma of no special type; CEUS, contrast-enhanced ultrasound

Recorded video loops were processed with Qlab® Quantification software (Philips). Both qualitative and quantitative analysis were employed for evaluation of blood perfusion of the lesions. The MicroVascular Imaging (MVI) software was used to assess the qualitative characteristics of lesions, an example of this method is shown in Fig. 1. The type of vascularization (peripheral or central), the perfusion homogeneity (homogenous vs. heterogenous) and the enhancement degree of lesions compared to the surrounding tissue (poor or absent vs. intermediate vs. rich) were assessed. For quantitative analysis, ROI was placed in the recorded video in the target area in order to analyze the changes in blood flow over time. Two square ROI of standard size 5 mm^2^ were drawn. The first ROI was placed in the target area of the highest enhancement of a lesion, the second, comparative, ROI was inserted into the surrounding breast tissue at least 1 cm away from a lesion. In the case of a large lesion, when the second ROI could not be placed in the same video loop, additional examination was performed in the same quadrant of the breast outside the observed lesion. The time-intensity curves (TI curves) generated from perfusion data (Fig. 2) were analyzed with a pre-defined software function gamma variate, fitting the saturation curve, which produced all required parameters of quantitative analysis in a short timeframe. These parameters were TTP (time to peak, s.), PI (peak intensity, dB), WIS (wash in slope, dB/s), AUC (area under curve, dB x s). Predefined motion compensation and background set were also applied to obtain these parameters. Motion compensation is an automatic function which detects slight movements in concordance with movements of ROI and eliminates their influence. The background set eliminates the effect of the initial non-zero setting of Time Gain Compensation before contrast administration and thereby eliminates false increase of absolute perfusion intensity. Thus, only the gain of the signal after the application of contrast agent is evaluated. Application examples are given in Fig. 3**.**

### Statistical analysis

To summarize the observed continuous (quantitative) parameters, basic descriptive statistics were used. Differences in these parameters according to the type of tumor were evaluated using the Mann-Whitney test. The categorical (qualitative) parameters were summarized using absolute and relative frequencies and compared by Fisher’s exact test.

The relationship between the malignant or benign type of lesion and the observed CEUS parameters was evaluated using a logistic regression model and described by the odds ratio (OR) with a 95% confidence interval (CI) for OR and a *p*-value corresponding to the significance of the respective regression coefficient.

## Results

### Patients and lesions characteristics

A total of 221 patients with 230 breast lesions were enrolled in this prospective study. 17 lesions found in 14 patients were excluded from the analysis (13 patients did not undergo recommended follow-up and 1 patient developed adverse reaction (vomiting) shortly after the contrast administration). Thus, 213 lesions were included in the analysis of the contribution of quantitative and qualitative CEUS parameters in differential diagnosis of breast lesions. In total, 146/213 (68,5%) lesions were benign and 67/213 (31,5%) malignant.

Basic patient and lesion characteristics are summarized in Table 1. Patients with a malignant tumor were older (median 65 years vs. 54 years) and their lesions were larger (median 17 vs. 10 mm). Predominantly higher grade tumors occurred in the group of malignant tumors (almost 50% of patients had grade 2 tumors and 37% of lesions were grade 3).

**Table 1:** Basic patient and lesion characteristics

Characteristics	Benign lesions		Malignant lesions	
	*n* = 146	%	*n* = 67	%
**Age** (years)				
median	54		65	
range	18–89		26–89	
**Lesion size** (mm)				
median	10		17	
range	3–34		5–50	
**Side**				
left	89	61%	27	40.3%
right	57	39%	39	58.2%
left and right	0	0%	1	1.5%
**Tumor Grade**				
1	–	–	9	13.4%
2	–	–	33	49.3%
3	–	–	25	37.3%
**Histology of benign lesions**	*n* = 74		–	–
fibrocystic disease	32	43%	–	–
benign proliferative breast disease	12	16%	–	–
fibroepithelial tumor	11	15%	–	–
intraductal proliferative lesion	10	14%	–	–
others	9	12%	–	–
**Histology of malignant lesions**	–	–	n = 67	
invasive carcinoma NST	–	–	40	60%
invasive lobular carcinoma	–	–	13	19%
others	–	–	14	21%

Abbreviations: *NST* invasive carcinoma of no special type

Of 146 benign lesion, 74 (74/146; 51%) were verified by a pathologist (66 cases after core-needle biopsy, 8 cases after surgical extirpation). The other lesions (72/146; 49%) were evaluated as BI-RADS category 3 and therefore were not histopathologically verified; no changes were detected during a follow-up over at least 2 years. Of the benign lesions, the most frequent was fibrocystic breast disease (FCD), which accounted for a total of 32/74 (43%) of histologically verified lesions.

Altogether, 67 lesions of breast cancer were examined and histopathologically validated. The most common histopathological type (40/67; 60%) was invasive carcinoma of no special type (NST), followed by invasive lobular carcinoma (13/67; 19%).

### CEUS parameters and type of tumor

The parameters of the quantitative and qualitative analysis of the CEUS of breast lesions are summarized in Table 2.

**Table 2:** Quantitative and qualitative parameters of breast CEUS according to the type of the tumor

	Benign lesions (***n*** = 146)		Malignant lesions (***n*** = 67)		***p***-value
**Quantitative parameters**					
**TTP** (s)					**< 0.001**
mean/ median/ range	29/ 25/ 4–152		20/ 18/ 4–70		
**WIS** (dB/s)					**< 0.001**
mean/ median/ range	0.13/ 0.04/ 0–1.64		0.24/ 0.14/ 0–1.63		
**PI** (dB)					0.105
mean/ median/ range	2.8/ 2/ 0.1–10.5		2.9/ 2.8/ 0–8.6		
**AUC** (dB.s)					0.711
mean/ median/ range	149/ 102/ 5–602		142/ 126/ 1–492		
**Qualitative parameters**	n = 146	%	n = 67	%	
**Type of vascularization**					0.117
peripheral	117	80%	47	70%	
peripheral + central	29	20%	20	30%	
**Perfusion homogeneity**					0.234
homogeneous	12	8%	2	3%	
heterogeneous	134	92%	65	97%	
**Enhancement degree**					**< 0.001**
poor/absent	71	48.6%	7	10.4%	
intermediate	47	32.2%	23	34.3%	
rich	28	19.2%	37	55.2%	

Abbreviations: *CEUS* contrast-enhanced ultrasound; *TI* time intensity curves; *TTP* time to peak; *WIS* wash in slope; *PI* peak intensity; *AUC* area under curve

Statistically significantly lower TTP values (on average by 9 s) and higher WIS values (on average by 0.11 dB / s) were observed in malignant tumors compared to benign tumors. A statistically significant difference was also found in the enhancement degree when a rich vascularity was detected in 55.2% of malignant lesions but only in 19.2% of benign lesions. There was no statistically significant difference in the character of the blood supply in the surrounding tissue of benign and malignant breast lesions.

The results of univariate logistic regression models quantifying the relationship between types of lesions (malignant, benign) and individual CEUS parameters are summarized in Table 3.

**Table 3:** Univariate and two multivariate logistic regression models for diagnosis of malignant lesion

	Univariate association with malignancy			Multivariate association with malignancy Model 1			Multivariate association with malignancy Model 2		
	*n* = 213	Crude OR (95% CI)	*p*-value	*n* = 213	adjusted OR (95% CI)	*p*-value	*n* = 213	adjusted OR (95% CI)	*p*-value
**Quantitative parameters**									
**TTP** (10 s)		0.65 (0.50–0.84)	**0.001**	213	0.78 (0.59–1.03)	0.076	–	–	–
**WIS** (1 dB/s)		6 (1.71–21)	**0.005**	–	–	–	213	1.6 (0.42–6.09)	0.489
**PI** (1 dB)		1.04 (0.91–1.19)	0.575	–	–	–	–	–	–
**AUC** (10 dB x s)		1.00 (0.97–1.02)	0.698						
**Qualitative parameters**									
**Type of vascularization**									
peripheral	164	1.00	ref.	–	–	–	–	–	–
peripheral + central	49	1.72 (0.89–3.33)	0.110	–	–	–	–	–	–
**Perfusion homogeneity**									
homogeneous	14	1.00	–	–	–	–	–	–	–
heterogeneous	199	2.91 (0.63–13.39)	0.170	–	–	–	–	–	–
**Enhancement degree**									
poor or absent	78	1.00	–	78	1.00	–	78	1.00	–
intermediate	70	4.96 (1.97–12.49)	**0.001**	70	4.1 (1.6–10.47)	**0.003**	70	4.83 (1.91–12.18)	**0.001**
rich	65	13.40 (5.35–33.59)	**< 0.001**	65	10.38 (4.04–26.72)	**< 0.001**	65	12.00 (4.57–31.52)	**< 0.001**

Abbreviations: *OR* odds ratio; *CI* confidence interval; *TI* time intensity curves; *TTP* time to peak; *WIS* wash in slope; *PI* peak intensity; *AUC* area under curve

A statistically significant relationship was found between the parameters TTP, WIS and enhancement degree. For every 10s increase in TTP the probability of malignancy decreased by 35%. On the other hand, for every 1 dB/s increase of WIS the probability of malignancy increased 6 times. Furthermore, the higher the enhancement degree the greater the risk of malignancy. A lesion with intermediate enhancement degree is 5 times more likely to be malignant than a lesion with poor or absent perfusion. A richly vascularized lesion is up to 13 times more likely to be malignant than a poorly perfused lesion.

Significant variables from univariate analysis were further analyzed in multivariate logistic regression with 2 models combining one qualitative and one quantitative parameter (Table 3). The first model included the parameters enhancement degree and TTP, the second model included the enhancement degree and WIS. Neither of the quantitative parameters (TTP or WIS) increased the accuracy of the enhancement degree in distinguishing between malignant or benign type of a lesion.

All above mentioned logistic regression models were compared using ROC curves, the results are given in Table 4. Sufficiently discriminatory models (AUC_ROC_ > 60%) used the parameters enhancement degree, WIS and TTP. The highest AUC was observed with the parameter enhancement degree (AUC_ROC_ = 74.7, 95% CI: 67.8**–**81.6**).** Higher sensitivity (89.6%) than specificity (48.6%) of this model indicates that it has a better ability to distinguish malignant tumors. The other two models with the parameters WIS (AUC_ROC_ = 69.8, 95% CI: 62.1–7.5) and TTP (AUC_ROC_ = 67.8, 95% CI: 60.2**–**75.5**)** are also more accurate in distinguishing malignant lesions. In the multivariate logistic regression models, the selected quantitative parameters did not significantly improve prediction of malignancy. The combination of these parameters with the enhancement degree slightly increased the area under the ROC curve (in case of TTP by 3.5%, in case of WIS by 1.0%) and increased specificity (ability to recognize benign tumors), while sensitivity (the ability to recognize malignant tumor) decreased.

**Table 4:** Logistic regression models

	ROC analysis of logistic regression models				
Univariate logistic regression models	AUC_**ROC**_ (%)	95% CI (%)	***p***-value	Senzitivity (%)	Specificity (%)
TTP	67.8	60.2–75.5	**< 0.001**	77.6	52.7
WIS	69.8	62.1–77.5	**< 0.001**	74.6	66.4
PI	56.9	49.0–64.8	0.105	85.1	37.7
AUC	48.4	40.3–56.5	0.711	92.5	18.5
Type of vascularization	55.0	46.5–63.5	0.242	29.9	80.1
Perfusion homogeneity	52.6	44.4–60.8	0.540	97.0	8.2
Enhancement degree	74.7	67.8–81.6	**< 0.001**	89.6	48.6
**Multivariate logistic regression models**	**AUC**_**ROC**_**(%)**	**95% CI (%)**	***p*-value**	**Senzitivity (%)**	**Specificity (%)**
**Model 1** (TTP + enhancement degree)	78.2	71.7–84.8	**< 0.001**	86.6	63.7
**Model 2** (WIS + enhancement degree)	75.7	68.5–82.8	**< 0.001**	79.1	70.5

Abbreviations: *AUC* area under curve; *CI* confidence interval; *TI* time intensity curves; *TTP* time to peak; *WIS* wash in slope; *PI* peak intensity

## Discussion

CEUS is currently a widely used diagnostic method allowing real-time evaluation of microvascular architecture. CEUS is mostly used to assess lesions of liver, kidneys and inflammatory bowel conditions. The possibility of incorporating CEUS into the diagnostic algorithm of breast lesions is still a subject of investigation. Malignant tumours in our study showed statistically significantly lower TTP parameters (sensitivity 77.6%, specificity 52.7%) and higher WIS values (sensitivity 74.6%, specificity 66.4%) than benign tumors. Enhancement degree also proved to be statistically well discriminating as 55.2% of malignant lesions had a rich vascularity (sensitivity 89.6% and specificity 48.6%). The combination of quantitative analysis parameters (TTP, WIS) with enhancement degree did not result in higher accuracy in distinguishing between malignant and benign breast lesions.

We expect that CEUS will become an effective tool in evaluation of breast lesions with unclear findings on conventional ultrasound, i.e. in distinguishing lesions BI-RADS category 3 and 4 [6]. Thus, CEUS may reduce the number of core-needle biopsies of benign lesions in the future [7], especially in older patients with higher body mass index larger maximal lesion diameter and distance to pappila [8]. Compared to magnetic resonance, CEUS is a relatively easily accessible, fast and cost-effective method well-suited to become a part of the diagnostic algorithm of breast examination before biopsy.

Newly formed tumor blood vessels are different from normal capillaries, they are characterized by irregular shape, abnormal calibre, fenestrated endothelium and formation of perivascular spaces. All of these changes lead to different perfusion, increased permeability and deregulation [9–11]. The expression of vascular endothelial growth factor (VEGF) and its receptor belongs among prognostic factors for breast cancer together with the tumor size and histological grade [12–14]. Generally, we can expect higher perfusion of breast lesions compared to the surrounding tissue and different characteristics of perfusion parameters according to the aggressiveness of the tumor. This knowledge has already been employed in the evaluation of Breast MRI. The study by Ricci [15] compared the results of the CEUS and MRI examinations and described CEUS as a reliable method for differential diagnostic algorithm as CEUS shows typical enhancement characteristics of lesions including perfusion curves which are comparable with MRI TI curves. Differences between benign and malignant breast lesions can also be found in unenhanced Doppler ultrasound. In our study, we observed a rich vascularization in 19.2% of benign lesions and in 55.2% of malignant lesions, based on MVI technology assessment. In some cases, benign and malignant lesions differ in other characteristics such as perfusion homogeneity and the type of vascular supply. Qualitative analysis of CEUS of malignant lesions was studied by Cao [16], the authors also associate the following characteristics with malignancy: perfusion defect, penetrating blood vessels, heterogeneous enhancement and centripetal enhancement. They concluded that these parameters can predict breast cancer prognosis in vivo.

In our study, differences in vascular perfusion kinetics were demonstrated based on TI curves. Significantly lower TTP values ​​and significantly higher WIS values ​​were associated with malignant tumors. These findings can be explained by earlier and faster onset of enhancement of malignant lesions. A similar conclusion can be found in Szabo’s study [11] which focused only on CEUS characteristics of verified breast cancer and demonstrated earlier peak enhancement (analogical to TTP and WIS parameters) and faster elimination of microbubbles in more aggressive forms of cancer associated with a poor prognosis. These findings may be explained by high occurrence of arteriovenous shunts in malignant tumors [17, 18]. In our study, evaluating the other qualitative (type of vascularization, perfusion homogeneity) and quantitative parameters (PI, AUC) did not significantly improve the ability to differentiate between benign and malignant lesions.

Qualitative, quantitative and combined analysis of CEUS of breast lesions was also studied by Wan et al. [19]. Their results show better diagnostic performance of qualitative and combined analysis than quantitative analysis, despite the fact that quantitative analysis appears at first sight to be more reliable due to its objectivity.

When assessing breast lesions, it is necessary to base the assessment not only on the results from the CEUS examination but on the conventional B-mode imaging as well. This subject has been studied in Du’s work [20], which states that the combined use of conventional B-mode sonography and CEUS provides greater diagnostic efficacy than either of these methods alone. The sensitivity of the combined examination is 81.8% and the specificity is 78.6%, these results are comparable to those achieved with MR.

In order to interpret the findings accurately [21], the main limitations of CEUS need to be acknowledged. It is a very operator-dependent technique therefore an experienced operator is needed. Timing of the examination is important, because the onset of perfusion depends on cardiac output. Difficulty may be caused by the variation in the level of basal perfusion between individual mammary glands. There may be changes depending on menstrual cycle which have to be considered, as it is in magnetic resonance examinations. Caution must be taken when compressing the target area with a probe, the neo-vessels of tumors are usually fragile and easily compressible, stronger compression would in consequence devalue the results. An obvious limitation of two-dimensional CEUS perfusion imaging is the acquisition of information from a single slice of tissue. A different approach would have to be chosen to examine the whole lesion (for example, to assess only qualitative parameters of images and to move the probe slightly while recording a video-loop). Also, there is the possibility of three-dimensional perfusion imaging, which has already been studied with positive results in the Jia et al. study [5].

The other methods of advanced ultrasound examinations such as elastography [22] should be consedered in ambiguous findings [23, 24], also considering the availability of pertinent ultrasound method as mentioned in introduction section.

## Conclusion

Contrast-enhanced ultrasound is a widely available, non-invasive and compared to magnetic resonance, less expensive method. Through qualitative and quantitative analysis, CEUS provides reproducible assessment of lesion vascularity and can also predict its type (benign or malignant). According to our results, qualitative analysis, particularly the description of enhancement degree, appears to be a more reliable assessment method, despite subjective evaluation. In the hands of experienced radiologists, CEUS, combined with conventional ultrasound and optionally with mammography, is an effective tool to ensure higher diagnostic accuracy and promises to reduce the number of core-needle biopsies of benign lesions in the future. In inconclusive findings, histopathological verification remains the method of choice.

## Data Availability

The datasets used and/or analysed during the current study are available from the corresponding author on reasonable request.

## Abbreviations

AUC: Area under curve
BI-RADS: Breast Imaging-Reporting and Data System
CEUS: Contrast-enhanced ultrasound
CI: Confidence interval
MVI: Microvascular Imaging
PI: Peak intenzity
ROI: Region of interest
TI: Time intensity
TTP: Time to peak
WIS: Wash in slope
